# 

**Published:** 1878-09-20

**Authors:** 


☞ All communications relating to the business of THE RECORD for the years 1877 and 1878, must be addressed DR. R. C. WORD, Managing Editor Southern Medical Record, Atlanta, Ga.

☞ Brief and practical communications are solicited on all subjects pertaining to medicine; also reports o[illegible]ses in practice.

☞ Send money by check, postal order or registered letter.

☞ Write your name, post-office, county and State plainly.

